# Orthodontic Implants: Concepts for the Orthodontic Practitioner

**DOI:** 10.1155/2012/549761

**Published:** 2012-11-11

**Authors:** Carlos Nelson Elias, Antônio Carlos de Oliveira Ruellas, Daniel Jogaib Fernandes

**Affiliations:** ^1^Biomaterials Laboratory, Military Institute of Engineering, 22290-270 Rio de Janeiro, RJ, Brazil; ^2^Department of Orthodontics, Federal University of Rio de Janeiro, 21941-617 Rio de Janeiro, RJ, Brazil

## Abstract

Orthodontic implants have become a reliable method in orthodontic practice for providing temporary additional anchorage. These devices are useful to control skeletal anchorage in less compliant patients or in cases where absolute anchorage is necessary. There are a great number of advantages in this new approach which include easy insertion, decreased patient discomfort, low price, immediate loading, reduced diameter, versatility in the forces to be used, ease of cleaning, and ease of removal. However, a proper management of the screws by the practitioner is necessary in order to increase the success rate of the technique. The purpose of this paper is to update practitioners on the current concepts of orthodontic implants and orthodontic mechanics.

## 1. Terminology

A wide range of devices may be implanted in and around the jaws therapeutically, accidentally, or for social reasons, many of which are not endosseous dental implants. The orthodontic implants are used for specific time periods and do not always have osseointegration. Other terms such as miniscrews, miniscrew implants, microscrews, microscrew implants, and temporary anchorage devices have been used. In the literature [1, 2] terms such as mini/microimplants and mini/microscrews are often used interchangeably. It is more suitable to refer by the “screw” terminology to a variety of internal fixation devices intended to aid in the alignment and stabilization of fractures to the skeletal system until healing has occurred.Screws are fabricated from stainless steel, titanium alloy (TiAlV, TiNbZr, TiMoZr), Cr-Co alloy, and rigid polyurethane. Dental implants are made from commercially pure titanium and Ti-6Al-4V ELI (Extra Low Interstitial).

There is no general agreement on the nomenclature. However, the standard ISO 16443 (Dentistry—Vocabulary of Oral Implantology) specifies terms and definitions for dental implants, instruments and accessories, and the most commonly used clinical terms in the field of dental implantology. In this standard, a dental implant is defined as a device especially designed to be placed within, through, or upon the bones of the craniofacial complex, the primary purposes of which are to support and to resist displacement of a dental prosthesis. According to this standard, the orthodontic implant is a device specially designed to be placed within, through, or upon the bones of the cranio-facial complex, the primary purpose of which is to provide anchorage for an orthodontic appliance. The purposes of orthodontic implants and dental implants are different. In this standard we do not have the terms or definitions of miniscrew and mini-implant. In the present work we will use the term orthodontic implant (OI).

## 2. Orthodontic Anchorage

**Figure 1:**
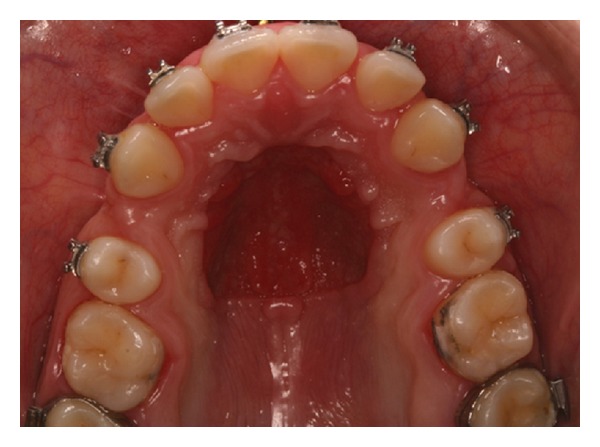
This photograph shows inaccuracies in the support structure due to a Nance button anchorage reinforcement appliance used for a long period.

Orthodontic implants have become broadly accepted as alternatives to extraoral devices in patients who either have insufficient dental support suitable for orthodontic anchorage or are not compliant in wearing extraoral devices. Orthodontic anchorage can be defined as resistance to unwanted tooth movement. During orthodontic treatments, different techniques can be devised and used to reinforce anchorage. Traditional biomechanical techniques, such as the use of extraoral anchorage by headgear or intraoral, one by bars, palatal/lingual arches or intermaxillary elastics, cannot effectively control anchorage, either due to lack of patient compliance or due to inaccuracies in the support structures (Figure 1).

The orthodontic anchorage can be classified according to the ratio of anterior retraction to posterior teeth protraction. Mild anchorage refers to slight retraction of anterior teeth while absolute anchorage means that the majority of space closure needs to be achieved by incisors retraction. Moderate anchorage entails a reciprocal management of anterior retraction and posterior protraction [3].

Traditionally, mechanical orthodontics principles are based on the support (anchorage) provided by the surrounding dental units from which reactive forces may result in anchorage loss (protraction of posterior teeth). In order to avoid undesirable dental movements, several anchorage situations require patient compliance, including headgear appliances. This subjection is substantially vulnerable to mechanical failure which results in an undesired orthodontic finish.

In order to improve anchorage, commercially pure titanium dental implants are increasingly used due to osseointegration. Although the absolute anchorage is achieved, limitations are present, such as longer healing time, difficulty of matching sites with implant sizes, the need for invasive surgery, cleaning problems, and, mainly, the difficulty of removing the implant after the orthodontic treatment is completed. Hence, in 1997, Kanomi [4] proposed the use of titanium orthodontic implants with 1.2 mm in diameter and 6 mm in length for orthodontic anchorage. Although not suitable for skeletal anchorage, these devices were effective for dental anchorage and provided enough stability for orthodontic purposes. 

## 3. Classification

**Figure 2:**
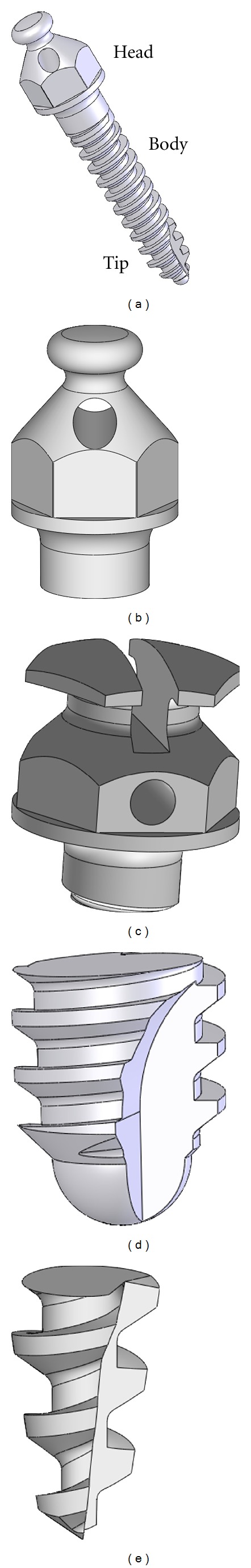
Typical orthodontic implants (a). Head type button (b). Head type bracket (c). Tip type self-tapping (d). Tip type self-drilling (e). (Courtesy of Marcos Joqueira, Conexão Sistemas e Próteses Co. Brazil.)

The orthodontic implants for anchorage (Figure 2) can be further classified according to:

- head design: button (Figure 2(b)) or bracket (Figure 2(c)),
- diameter: from 1.2 to 2.0 mm,
- length: from 5.0 to 12.0 mm,
- body design: tapered or cylindric,
- transmucosal profile: from 0.0 to 3.0 mm,
- insertion technique: self-tapping (Figure 2(d)) or self-drilling (Figure 2(e)),
- thread orientation: left or right drilling insertion,
- alloy used for fabrication: titanium alloy ASTM grade 5 or stainless steel.

**Figure 3:**
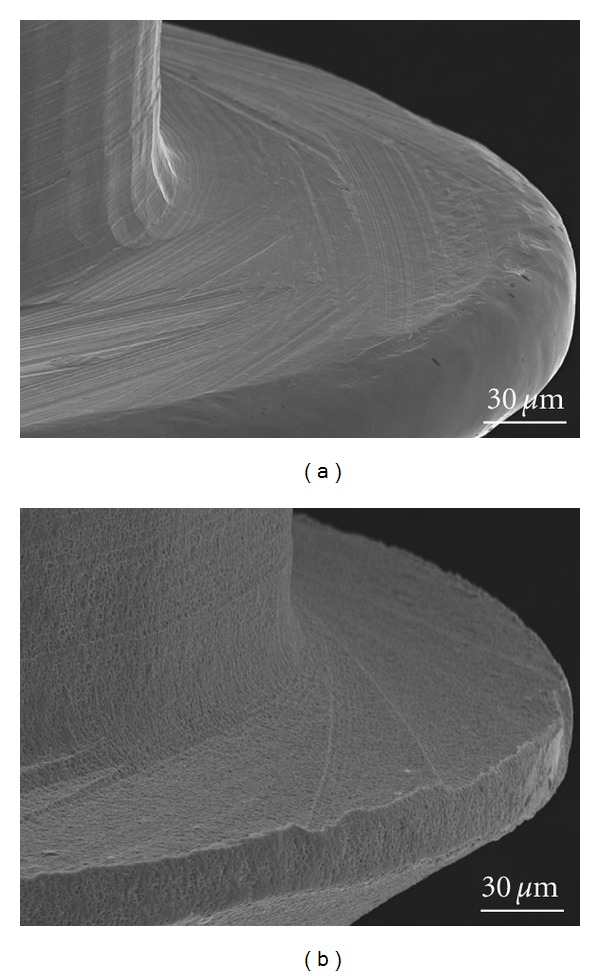
SEM images of OI surface morphology. (a) OI without surface treatment. (b) OI with acid surface treatment. Notice the grooves in the OI surface of the thread when compared with another which received acid surface treatment.

Typically, the orthodontic implants are made of titanium alloy ASTM grade 5 (Ti-6Al-4V) and are not subjected to surface treatment (Figure 3(a)). The machined orthodontic implants without surface treatment have tool marks and grooves. The surface of the dental implant is treated, which increases its roughness and osseointegration. The implant surface roughness governs cell interactions, allowing adhesion, proliferation, and differentiation. An adequate implant surface increases the bone-implant contact (BIC) and makes the orthodontic implants most suitable for immediately loading [4]. However, the OI surface treatment increases the torque needed for device removal [5].

## 4. Orthodontic Implant Applications

By providing temporary skeletal anchorage, orthodontic implants have several applications. These devices can be employed to allow corrections in:

- vertical dimension: molar intrusion in anterior open bite cases, lower molar intrusion in high angle patients, incisor intrusion in deep bite with gingival display, and undesirable occlusal plane angulation;
- anteroposterior dimension: full-step class II malocclusions associated with unpleasant profile, biprotrusive patients which are unwilling to cooperate with headgear or intermaxillary mechanics, cases where full canine anterior movement is necessary in order to substitute a missing lateral incisor (agenesis), and molars; premolars protraction cases are also indicated when great space closure is necessary;
- prosthetic cases which need single tooth movement without a complete fixed appliance.

## 5. Clinical Procedure for Orthodontic Implant Insertion

The size and design of the orthodontic implants should be compatible with the quantity and quality of bone available at the installation site and depends on the orthodontic mechanics demanded and on how long the orthodontic implant will remain in situ [3].

**Figure 4:**
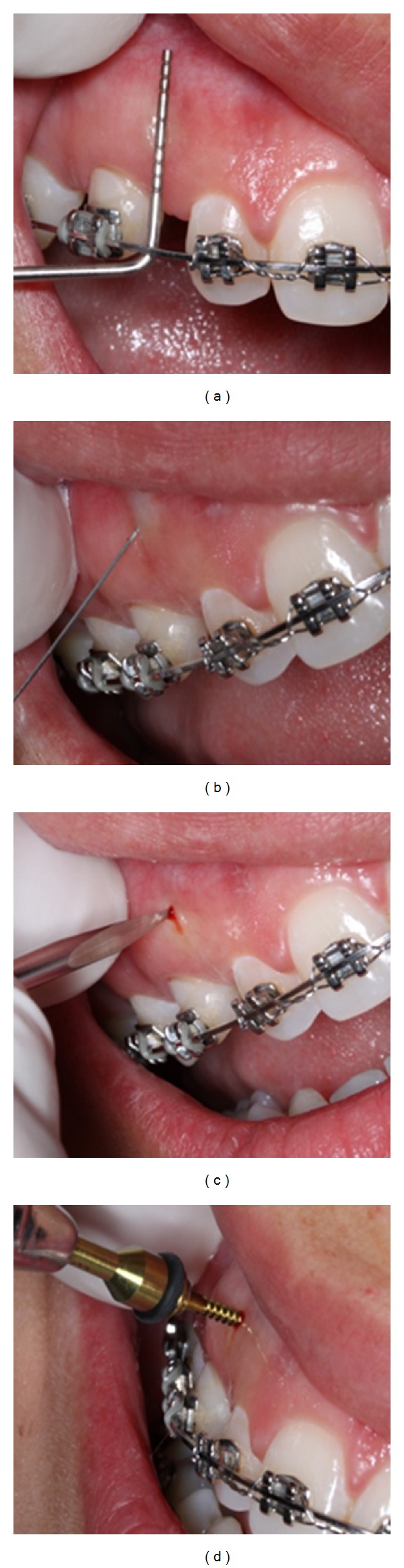
Self-drilling orthodontic implant procedure: (a) marking the space between adjacent roots; (b) punch soft tissue anesthesia; (c) pilot drilling with a lance (d) perpendicular insertion of OI without lateral movements or excessive torque.

Figure 4 shows an example of the insertion phases of OI. Only three or four steps are needed for OI insertion:

- determination of the height of the installation site with a probe, taking into account the position of adjacent tooth roots and other anatomical structures such as inferior alveolar nerves, arteries, veins, mental foramen, and nasal cavity;
- soft tissue anesthesia;
- pilot drilling with a lance;
- insertion of OI.

The insertion of orthodontic implants can be performed with or without prior drilling, depending on the insertion site and the tip of the orthodontic implant. To reduce the fracture risk, prior drilling is necessary in the case of high bone density and large cortical thickness. An example is the insertion of OI in ancient edentulous areas. Commonly, drilling is performed at the same hole of anesthesia. The orthodontic implant placement procedure is usually performed by the orthodontist with high success rates [6], since it is a minimally invasive procedure.

Normally, the OI is inserted about 2 mm from the tooth root [7]. Alves et al. [8] found that, although the OI is meant to provide stable skeletal anchorage, it has some degree of displacement (≤0.78 mm).

According to volumetric tomography evaluations, the safest insertion sites in maxilla are the anterior and apical region. In the palate, midpalate, and a site distant 6 to 9 mm from the incisive foramen are suitable for implantation of small length (ranging from 4 to 6 mm) orthodontic implants. In the mandible, the safest area is between first and second premolars and the same site between molars. Factors such as the type of desired movement, biomechanics, distance between roots, attached soft tissue, palate bony height, torque, moment, and bone density should be also analyzed when choosing the site of installation [9].

**Figure 5:**
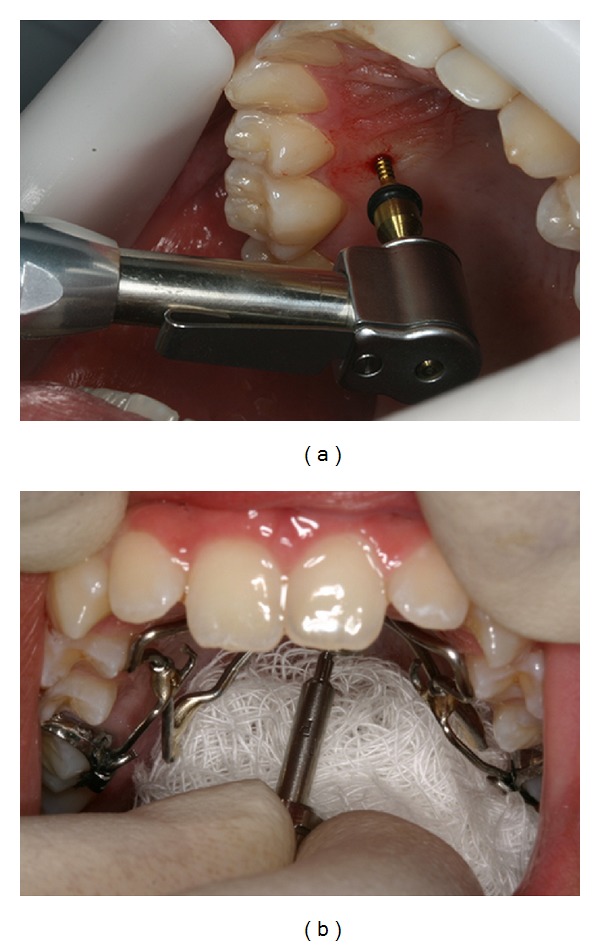
OI installation with the handpiece (a). OI insertion in the palate with a digital key and protection of the throat with gauze (b).

The insertion of OI in the palate (Figure 5) may be performed with the digital key or with angle guide. It is recommended to protect the patient's throat with gauze to prevent injuries (Figure 5(b)).

**Figure 6:**
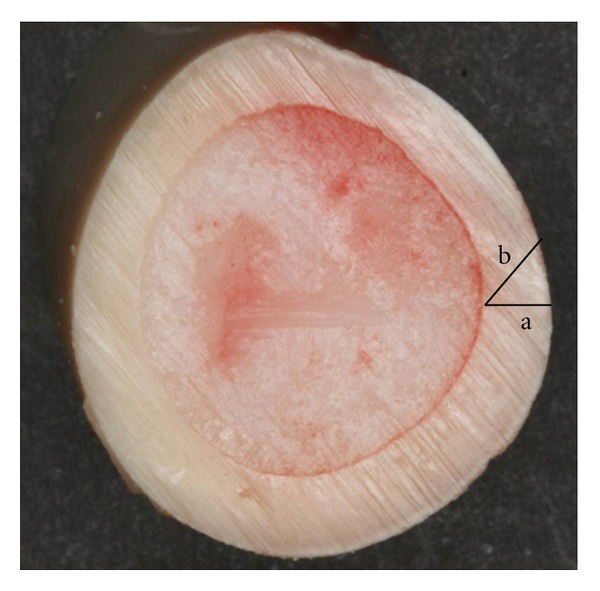
Different directions for OI installation. (a) Length of bone contact with OI inserted perpendicular to the cortical bone and (b) length of bone contact with OI inserted tilted with respect to cortical bone.

Orthodontic implants may be placed under an angulation between 10° and 20° and maybe up to 45°. In maxilla, particularly, a 30° to 40° angulation to the long axes of adjacent teeth and a 10° to 20° angulation in the mandible are recommended to avoid dental injuries. Besides, this angle increases the area of bone contact and ensures greater primary stability.  Figure 6 shows that changes in OI angulation increase the contact length between OI and cortical bone.  Table 1 shows the influence of angulation of the orthodontic implant on the interface OI-bone contact. Lateral movements should be avoided during installation. 

Another important factor for the successful installation of the orthodontic implants is the control of the insertion torque. An ideal torque should be large enough to provide sufficient primary stability yet low enough to maintain the vitality of the surrounding tissues responsible for the healing process which results in secondary stability [10]. It is recommended to use a torque wrench attached to a key insertion coupled with a torque limiter. The insertion and removal torques depend on the contact area between the implant surface and the bone, which is directly proportional to the length of insertion and the cube of the radius. For example, when the length of the orthodontic implant increases to 0.5 mm, the OI-bone contact length increases 33% and insertion area also increases by 33%. However, when the diameter of orthodontic implants increases from 1.5 mm to 2.0 mm, the diameter increases 33% but the MI-bone surface contact increases by 77.8%. The average torque to insert an orthodontic implant with 2.0 mm diameter (23.2 N·cm) is 84.1% higher than the torque to insert the 1.5 mm (12.6 N·cm). The insertion torque of orthodontic implants with 1.5 mm in high bone density is closer to the fracture torque and requires special care. This has clinical implications for the inclusion of OI in the mandible, where the higher insertion torque due to increased bone density may negatively affect the surrounding tissues. A OI placed with excessive torque might show adequate primary stability but over time would loosen and fail. It is also essential that anesthesia be applied only on superficial tissues. The patient feedback from discomfort can alert practitioner before the implant penetrates sensitive structures and may prevent irreversible damage during implant insertion.

Kim et al. [11] inserted 32 orthodontic implants in two beagle dogs and analyzed the influence of the use of prior drilling of the alveolus for placement of the OI. He found that devices inserted without prior drilling have higher anchorage and primary stability. Implants with larger diameters and self-tapping can increase the possibility of damage of cortical bone, which can affect bone remodeling, and stability [12]. Thus, it is recommended prior to drilling for insertion of self-tapping OI. This drilling is meant only to overcome the cortical thickness, since a true pilot hole extending into the bone the entire length of the implant is not necessary.

The stability of orthodontic implants is associated with several factors such as patient age and gender, the density, and thickness of cortical bone, OI threads design, immediate loading, and oral hygiene [13, 14].

## 6. Primary Stability

**Figure 7:**
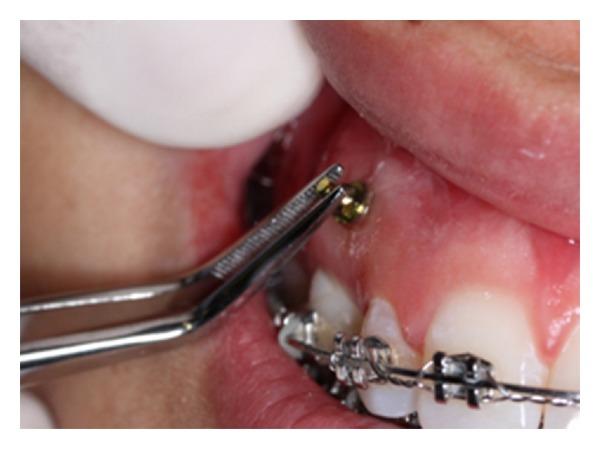
Primary stability evaluation with a tweezer immediately after implant insertion.

Primary stability (PS) or initial stability refers to the mechanical stability in the bone immediately after OI insertion. It is a prerequisite for healing, constituting one of the most important factors for the success rate of OI. PS is a function of the implant diameter, the implant length, the number, and design of threads, the cortical thickness, and the cortical bone density. It can be assessed indirectly by the insertion torque. After installation, the initial stability is tested by attempting to move it with a tweezer (Figure 7). The OI should not show signs of mobility. The success rate is assessed during the first four months of installation [13, 14].

The primary stability of orthodontic implants should be analyzed in terms of tensile, compressive and shear stress, the intensity of the applied forces, and the deformation of the surrounding structures. Shear stress is the most critical loading of the bone-OI interface. After OI insertion, the preload induces compression forces on the thread surface which tend to maintain OI stability.

Considering that orthodontic loads are applied perpendicular or angled, the removal resistance is greater than a simply pull-out test [15]. The orthodontic forces are between [16] 0.3 and 4.0 N, smaller than the OI tensile strength. The exact relationship between the applied force in the axial direction of the OI and the applied force in the tangential direction remains unknown.

**Figure 8:**
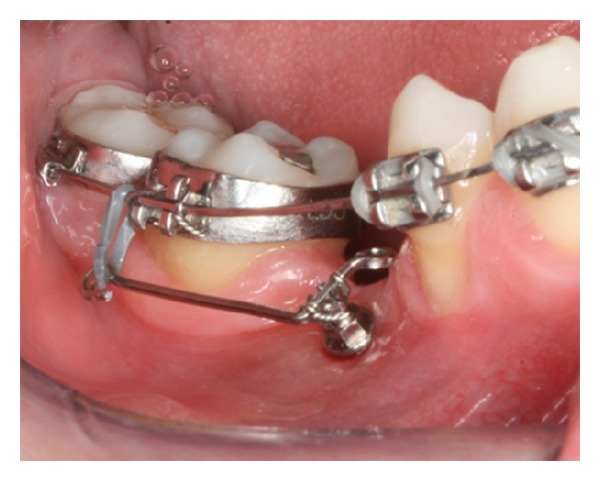
Counterclockwise orthodontic implant thread with bracket head.

Increased torsional stress during implant placement generates shear stress which can lead to bending and fracture of the implant. The maximum shear stresses are found in implants with bracket head during loading. If the implant become loose, a twist is necessary in order to allow activation of the insertion instead of its removal.  Figure 8 shows the application of a reverse threaded OI (left), allowing the activation of the intrusion of the posterior segment without the occurrence of the rotation in the opposite direction. The device would not become loose since the reactive force in OI tightens the device. Note that an excessive reactive force should be avoided to prevent the development of microcracks in the peri-implant bone that may affect the implant stability. 

Another important factor to prevent OI failure is control of compressive stress in the soft tissue during implant insertion. The implant platform may rest above the gingival tissue without penetrating it. The correct choice of the transmucosal profile ensures that the implant head will not penetrate the alveolar bone.

Orthodontic implants with large diameter and conical shape can induce excessive compression of the cortical bone with higher insertion torque [17]. This can cause microdamage to the cortical bone in the form of cracks and possible fracture. The accumulation of minor damage can produce local ischemia, bone necrosis, bone remodeling, and premature loss of the OI.

## 7. Complications

**Figure 9:**
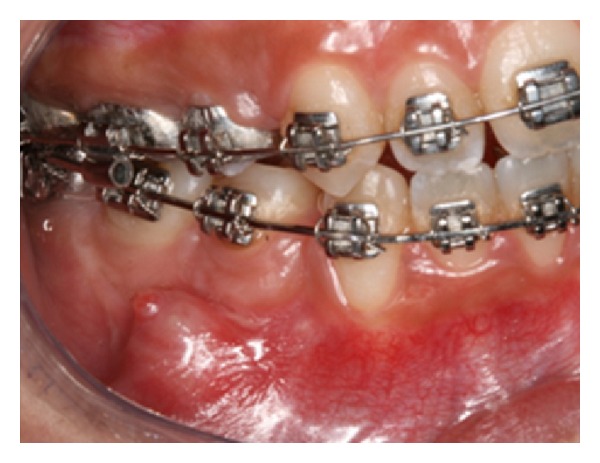
Soft tissue coverage of the orthodontic implant head.

Possible complications after OI insertion are inflammation of the soft tissue around the OI, difficulty inserting the elastic chain because of soft tissue covering the head of the OI (Figure 9), loss of stability (discussed above), and fracture.

**Figure 10:**
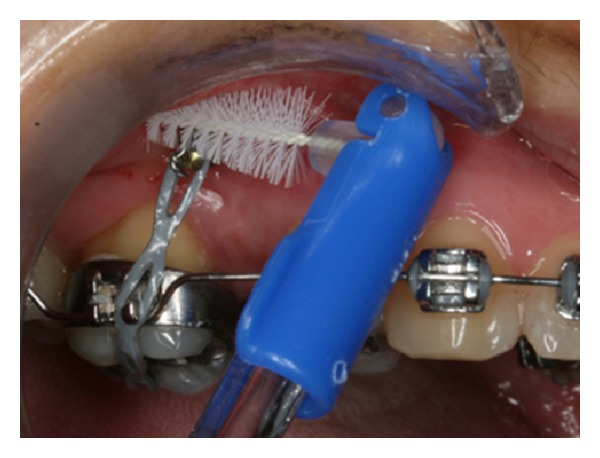
Interdental brush dipped in 0.12% chlorhexidine during OI hygiene control.

Hygiene control is essential to the success of orthodontic implants. It helps to prevent peri-implantitis or the inflammation of the tissues around the orthodontic implant. It is recommended that the implant site be cleaned with an interdental brush dipped in 0.12% chlorhexidine (Figure 10).

The main risk of OI fracture occurs during insertion and is mainly a result of excessive torque. The development of the sense of touch, feeling the resistance to insertion, appropriate selection of the site, the use of digital twist, previous perforation of the cortical (if necessary, in edentulous cortical areas), attention to pain symptoms, and signs of excessive increase of resistance to the penetration of the OI are essential to prevent fractures. Fracture of the orthodontic implant during removal may occur if the neck of the orthodontic implant is too narrow. It is advisable to use orthodontic implants with a minimum diameter of 1.6 mm and 8.0 mm of length when placed in dense cortical bone.

**Figure 11:**
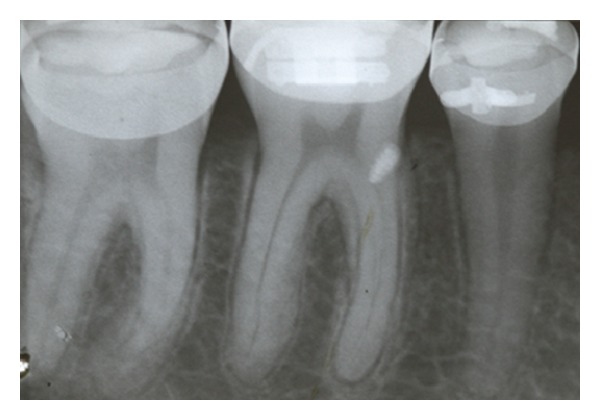
Fractured OI tip that was kept buried.

If OI fracture occurs, two procedures can be done: (a) removing the fractured part or (b) keeping the fractured part buried (Figure 11), since it is not necessary to move the tooth in the area.

## 8. Orthodontic Implant Removal

Removal of the OI is done without anesthesia and twisting in the opposite direction to that used in the insertion. Therefore, it is recommended to note the type of orthodontic implant thread used (right or left) to prevent fracture during removal.

The removal torque is always less than the insertion one [6, 18]. In vitro tests [18] showed that the removal torque (5.4 N·cm) of orthodontic implants 2.0 mm from of the tibia of rabbits was 44.5% lower than the insertion torque (9.6 N·cm). In the same test, the removal torque of OI from bovine cortical (6.8 N·cm) of MI 1.5 mm was 45.6% lower than the insertion torque (12.6 N·cm).

Histological analyses showed that when the OI loading is made immediately after insertion, osseointegration in the OI-bone interface is smaller than when loading is delayed. On the other hand, early loading did not compromise the stability of the orthodontic implants during clinical treatment [2]. Some authors think this phenomenon is favorable because it facilitates the removal after treatment [19, 20].

## Figures and Tables

**Table 1:** Influence of the angulation of the orthodontic implant and the OI-bone interface contact (BIC).

Cortical thickness	Angulation	5°	10°	15°	20°	25°	30°	35°	40°	45°
2.0 mm	OI-bone contact length (mm)	2.01	2.03	2.07	2.13	2.21	2.31	2.44	2.61	2.83
	% increase BIC	0.38	1.54	3.52	6.41	10.33	15.45	22.05	30.50	41.37

2.5 mm	OI-bone contact length (mm)	2.51	2.54	2.59	2.66	2.76	2.89	3.05	3.26	3.53
	% increase BIC	0.38	1.54	3.52	6.41	10.33	15.45	22.05	30.50	41.37
